# Heterogeneity of executive functions among preschool children with psychiatric symptoms

**DOI:** 10.1007/s00787-019-01437-y

**Published:** 2019-11-11

**Authors:** Sini Teivaanmäki, Hanna Huhdanpää, Noona Kiuru, Eeva T. Aronen, Vesa Närhi, Liisa Klenberg

**Affiliations:** 1 Department of Psychology, University of Jyväskylä, P.O. Box 35, 40014, Jyvaskyla, Finland; 2 Niilo Mäki Institute, P.O. Box 35, 40014 Jyvaskyla, Finland; 3 Laboratory of Developmental Psychopathology, Pediatric Research Center, Child Psychiatry, Biomedicum Helsinki, P.O. Box 63, 00014 Helsinki, Finland; 4 University of Helsinki and Helsinki University Hospital, Children’s Hospital, Child Psychiatry, Puistosairaala, P.O. Box 281, 00029 Helsinki, Finland; 5 Department of Education, University of Jyväskylä, P.O. Box 35, 40014 Jyvaskyla, Finland; 6 Department of Psychology and Logopedics, University of Helsinki, P.O. Box 21, 00014 Helsinki, Finland

**Keywords:** Executive functions, Preschool, Internalizing symptoms, Externalizing symptoms, Psychopathology

## Abstract

The aim of the present study was to investigate associations between internalizing and externalizing symptoms and deficits in executive functions (EF) as well as to examine the overall heterogeneity of EFs in a sample of preschool children attending a psychiatric clinic (*n* = 171). First, based on cut-off points signifying clinical levels of impairment on the parent-completed Child Behavior Checklist (CBCL), children were assigned into groups of internalizing, externalizing, combined or mild symptoms and compared to a reference group (*n* = 667) with regard to day care teacher ratings of EFs on the Attention and Executive Function Rating Inventory-Preschool (ATTEX-P). Second, latent profile analysis (LPA) was employed to identify distinct subgroups of children representing different EF profiles with unique strengths and weaknesses in EFs. The first set of analyses indicated that all symptom groups had more difficulties in EFs than the reference group did, and the internalizing group had less inhibition-related problems than the other symptom groups did. Using LPA, five EF profiles were identified: average, weak average, attentional problems, inhibitory problems, and overall problems. The EF profiles were significantly associated with gender, maternal education level, and psychiatric symptom type. Overall, the findings suggest that the comparison of means of internalizing and externalizing groups mainly captures the fairly obvious differences in inhibition-related domains among young psychiatric outpatient children, whereas the person-oriented approach, based on individual differences, identifies heterogeneity related to attentional functions, planning, and initiating one’s action. The variability in EF difficulties suggests that a comprehensive evaluation of a child’s EF profile is important regardless of the type of psychiatric symptoms the child presents with.

**Electronic supplementary material:**

The online version of this article (10.1007/s00787-019-01437-y) contains supplementary material, which is available to authorized users.

## Introduction

According to a contemporary definition, EFs include basic functions related to inhibition of responses and distracting stimuli, working memory, and flexible shifting of attention or response-set [1, 2] as well as more complex processes such as planning and use of strategies [3, 4]. EF difficulties are often present in children with different kinds of psychiatric problems [5–8]. Already in the preschool period, EF difficulties are common among young children referred to psychiatric care [9]. Previous studies examining the link between EFs and psychiatric symptoms have provided inconsistent findings and the majority of studies have focused on older children. In addition, only a few studies have examined the heterogeneity of EFs in mixed clinical groups on the level of individual variation instead of averaging across diagnostic/symptom groups.

EFs are known to follow a protracted course of development that parallels the relatively slow maturation of the prefrontal cortex [10], an essential part of the neuronal circuitry responsible for EFs. The basic forms of EF, particularly inhibition and working memory, start to develop in infancy [11]. Especially, the preschool period (roughly the ages of 3–6 years) is characterized by rapid development of EFs [11, 12]. During this time, gender differences are often evident, as girls tend to be ahead of boys in the development of EFs [13, 14]. Environmental factors also contribute to the development of EFs: especially, higher parental education and socioeconomic status have been associated with better EFs [14–16]. EFs are crucial for adjustment across all aspects of life. For children, EFs are important for school success [17, 18] and socioemotional competence [19]. They are also predictive of many outcomes, such as health and personal finances, later in life [20].

EF difficulties can be assessed with performance-based tests and behavioral rating scales. Performance-based tasks are administered under highly standardized conditions and yield information about the cognitive capacities related to EFs, while behavioral measures are based on observations of the child’s EF behaviors in daily situations. Although the typically used performance-based measures may give a detailed account of the child’s EF capacities, they do not correspond to the multifaceted and dynamic nature of real-world situations [21]. Thus, in addition to performance-based measures, rating scales should be used to provide clinical indicators of the child’s functional ability related to EF competence and difficulties.

Typically, the relations between EF difficulties and psychiatric symptoms have been examined on the level of different diagnostic or externalizing/internalizing symptom groups. Externalizing symptoms refer to problems directed primarily outwards and involving conflict with others, such as aggression, conduct problems, and hyperactivity [22]. Most studies examining EF deficits related to externalizing symptoms in school-aged children have used performance-based measures. In these studies, deficits in inhibition, working memory and set shifting have consistently been found [23–26]. Parent and teacher ratings of EFs in school-aged children with externalizing symptoms have generally revealed wide-ranging difficulties, with an emphasis on difficulties in inhibition and working memory [15, 27, 28]. Accordingly, preschool children with externalizing symptoms have been found to have inhibitory deficits and, although somewhat less consistently, deficits in working memory and set shifting when examined using performance-based measures [7, 29]. Parent [30, 31] and teacher ratings [32, 33] of EFs have revealed broad difficulties in the everyday environment for these young children.

Internalizing symptoms refer to inward-directed problems, such as anxiety, depression, withdrawal, and somatic complaints [22]. A limited amount of studies has examined EFs in children with internalizing symptoms/disorders. In a meta-analysis concerning depressed children and adolescents, impairments in interference control, planning, working memory, shifting, and phonemic and semantic verbal fluency were found [8]. Recent empirical evidence indicates that a deficit in cognitive flexibility, referring to the ability to shift attention and response-set, may specifically relate to internalizing symptoms [34]. However, many studies have not found EF deficits in depressed children and adolescents [35], and the extent to which the variability reflects methodological differences in sample selection, inclusion criteria, and EF tasks is unclear.

The relations between EFs and internalizing symptoms among preschool children are even less studied. Skogan et al. [36] found broad EF difficulties in 3-year-old children with an internalizing disorder (anxiety) when using the Behavior Rating Inventory of Executive Function–Preschool Version (BRIEF-P) to assess EFs. Eisenberg et al. [32] utilized both performance-based and rating scale measures with multiple informants in examining executive control in 4–8-year-old children. They reported that the children high in internalizing symptoms were rated as less impulsive and lower in attentional control than the control children, but similar with regard to inhibitory control. Finally, some recent longitudinal studies have found that preschool EF difficulties, especially in inhibition and flexibility, are related to internalizing symptoms in the elementary school years [37, 38].

The conflicting evidence on EF dysfunction in children with internalizing and externalizing symptoms points towards the heterogeneity of EF abilities within these clinical groups and even within single disorders. Person-oriented methods, such as cluster analysis or latent profile/class analysis, provide a useful approach in such instances by allowing the empirical identification of distinct subgroups based on different indicators, such as EF abilities. In contrast to the variable-oriented approach, the focus is on the individual instead of the group and on the configuration of information instead of the single variable representing a given construct [39]. The theoretical roots of the person-oriented approach can be found in the holistic–interactionist paradigm formulated by Bergman and Magnuson [40], which highlights the importance of studying individuals as organized wholes based on their unique patterns of characteristics. The basic tenet is that, despite the structure and dynamics of behavior being partly unique to individuals, there is still lawfulness to development, and often only a rather small number of typical patterns is enough to describe it adequately [40]. From a methodological perspective, the person-oriented approach may allow avoiding the pitfalls of data aggregation that often do not do justice to the individual nor to the possible subpopulations within the sample [41].

Only a few studies have taken a person-oriented route and addressed the heterogeneity of EFs by identifying subgroups within samples of children with psychiatric symptoms. Kavanaugh et al. [42] examined the presence of neurocognitive subgroups within a sample of child psychiatric inpatients using cluster analysis. Their study included measures of EFs as well as other cognitive functioning. Four subgroups—intact, global dysfunction, organization/planning dysfunction, and inhibition-memory dysfunction—were found. Using BRIEF scales and the Statue subtest from NEPSY as EF indicators, Dajani et al. [43] identified average, above average and impaired subgroups of EFs in a sample consisting of typically developing children and children with neurodevelopmental disorders. They concluded that the nature of EFs is dimensional in children, because no differences in strengths and weaknesses between the subgroups were found. Therefore, it remains uncertain whether subgroups displaying not only quantitative, but also qualitative differences in EFs can be found in clinical groups of children via person-oriented methods.

The aim of the present study was to investigate inter child variability in EF difficulties among clinically referred children. This was done in two stages. First, we followed a traditional group comparison approach by examining the EF difficulties of children classified into groups according to their level of internalizing and externalizing symptoms. We predicted that the groups with mainly externalizing and both externalizing and internalizing symptoms would have elevated scores (indicating more problems) in all EF domains in comparison with controls. Due to some previous studies suggesting that particularly flexibility difficulties may be closely related to internalizing symptoms, we expected that the internalizing group would have more problems than the reference group in, at the least, shifting attention. The externalizing group was expected to have more difficulties than the internalizing group in, at the least, impulsivity and motor hyperactivity. The aim of the second stage was to derive subgroups of children with distinct EF profiles based on individual-level variation in EFs. Because no previous study that we are aware of has investigated EF profiles in a mixed clinical sample of preschool children, we took an exploratory approach without specific hypotheses about the outcome. Finally, subgroup differences in age, gender, maternal education level, and internalizing/externalizing symptoms were investigated.

## Methods

### Participants

The clinical group consisted of children recruited from two psychiatric outpatient clinics evaluating and treating preschool children at Helsinki University Hospital, Child Psychiatry Unit. The data were collected between March 2015 and May 2017. Inclusion criteria were (a) child’s age between 4 and 7 years, (b) Finnish-speaking parents, and (c) child attending day care. Overall, 315 patients visited the two clinics during data collection, and 252 of them met the inclusion criteria and received the Attention and Executive Function Rating Inventory-Preschool (ATTEX-P) and CBCL questionnaires. Of them, 171 (67.8%) families returned both the study questionnaires. Due to lacking information about the non-participants, we were unable to perform a direct comparison between the participants and non-participants. However, the characteristics of the present sample were in line with the previous reports indicating high rates of comorbidity, a higher prevalence of boys than girls, and an overrepresentation of low maternal education among pre-school children referred to psychiatric care [44, 45]. The present sample was heterogeneous in terms of diagnoses: 39 (22.8%) children were diagnosed with ADHD, 29 (17.0%) children were diagnosed with either conduct disorder or oppositional defiant disorder, and diagnoses for other neurodevelopmental disorders, such as autism spectrum, learning, speech, and motor system disorders were also frequent (*n* = 34, 19.9%) [9]. 34 (19.9%) children had at least one Z-diagnosis describing psychosocial stress. In addition, 69 (40.4%) children had an unspecified neurodevelopmental diagnosis (F88 or F89), reflecting the fact that the psychiatric evaluation was not yet completed.

The reference group consisted of children who took part in the ATTEX-P [46] standardization study between August 2014 and May 2015. The data were collected from 28 day care units in a medium-sized city in the southern part of Finland. Inclusion criteria were (a) age between 4 and 7 years and (b) Finnish-speaking parents. Families delivered the ATTEX-P questionnaires to the day care units, and the questionnaires of 709 children were returned. The reference group was well representative of the Finnish population in terms of children’s gender and mothers’ educational-level distributions [47, 48].

Of the 880 participants, 8.3% had one or more missing observations on the ATTEX-P. In the clinical sample, a maximum of one value per participant was missing on any given scale, and all of the missing values were imputed by calculating the participant’s mean value for the scale items. The missing values for maternal education level (*n* = 3) in the clinical sample were replaced with the mode value of maternal education level within the participant’s respective symptom group. In the reference sample, participants with any missing values on the ATTEX-P (*n* = 24) or maternal education level (*n* = 19) were omitted from the analyses. These procedures resulted in a final sample of 838 participants, 667 in the reference group and 171 in the clinical group.

### Measures

#### Executive functions

The ATTEX-P [46] is a 44-item rating scale designed for assessing EF behavior of children aged 4–7 years in a day care environment. ATTEX-P is an adaptation of the ATTEX rating scale for school-age children [15] and covers a wide range of behaviors reflecting both basic and complex EF processes. The day care teacher rates the frequency of EF difficulties on a three-point scale (0 = not a problem, 1 = sometimes a problem, and 2 = often a problem). The questionnaire yields a total score as well as scores for nine clinical subscales: (1) distractibility (5 items), (2) impulsivity (10 items), (3) motor hyperactivity (5 items), (4) directing attention (5 items), (5) sustaining attention (4 items), (6) shifting attention (4 items), (7) initiative (3 items), (8) planning (3 items), and execution of action (5 items). Higher scores on the scales indicate more problems. The total score and the subscales have demonstrated good internal consistency (ranging from 0.73 to 0.94), test–retest reliability (ranging from 0.81 to 0.94), and convergent validity (correlations with EF items in a school readiness questionnaire ranging from 0.49 to 0.75) [46]. Total or scale scores at or above the 90th percentile are considered to indicate clinically relevant deficits in EF behavior.

#### Emotional and behavioral problems

Parent ratings of the child’s emotional and behavioral problems on the Child Behavior Checklist/1.5–5 **(**CBCL) [22] were used for grouping participants into subgroups according to externalizing and internalizing symptoms. CBCL is a parent-report form of a widely used questionnaire measuring children’s behavioral and emotional problems. The form contains 99 problem items rated on a three-point scale (0 = not true, 1 = somewhat or sometimes true, 2 = very true or often true). The questionnaire has demonstrated good reliability and validity [22] as well as generalizability across 23 societies [49]. The broadband internalizing problem scale consists of the following four symptom scales: emotionally reactive; anxious/depressed; somatic complaints; and withdrawn. The broadband externalizing scale contains the remaining symptoms scales: attention problems and aggressive behavior.

#### Background information

Information on age, gender, and maternal education level was collected from parents via a short questionnaire.

### Data analyses

#### Symptom group comparisons

For the symptom group comparisons, subgroups of children from the clinical sample were formed based on parent reports on the CBCL internalizing problems and externalizing problems scales. First, *T* scores of the raw scale scores were computed using ADM (9.1) scoring software. Children whose score reached the clinically significant problem level (*T* score > 63) on the Internalizing Problems or Externalizing Problems scale, but not on both scales, were included in the groups of children showing either internalizing symptoms (INT) or externalizing symptoms (EXT). Children with *T* scores greater than 63 on both scales were included in the combined group (COMB), and children with *T* scores below 63 on both scales were included in a group showing mild symptoms (MILD) (Table 1).

**Table 1:** Descriptive variables concerning the symptom groups and the reference group

	INT	EXT	COMB	MILD	REF
Sample size, *n*	24	21	60	66	667
Age in years, *M* (SD)	5.7 (0.6)	5.8 (0.6)	5.6 (0.7)	5.8 (0.8)	6.0 (0.7)
Gender					
Male (%)	14 (58.3)	17 (81.0)	48 (80.0)	43 (65.2)	341 (51.1)
Female (%)	10 (41.7)	4 (19.0)	12 (20.0)	23 (34.8)	326 (48.9)
Mother’s education					
Low (%)	7 (29.2)	13 (61.9)	34 (56.7)	38 (57.6)	273 (40.9)
High (%)	17 (70.8)	8 (38.1)	26 (43.3)	28 (42.5)	394 (59.1)

*INT* children with internalizing symptoms, *EXT* children with externalizing symptoms, *COMB* children with combined symptoms, *MILD* children with mild symptoms, *REF* reference children

Using SPSS 24, the symptom groups were compared to the reference group in EF variables. Overall group differences on the ATTEX-P total score were analyzed with ANCOVA, and differences in the scale scores were examined with MANCOVA, followed by separate ANCOVAs for the scale scores and pairwise comparisons for group contrasts. A Bonferroni-corrected significance level *p* < 0.005 was applied in the pairwise comparisons to account for the 10 comparisons. Variables representing age, gender, and maternal education level were included as covariates in all analyses. The effect sizes are reported as partial eta squared ($\eta_{\text{p}}^{2}$; small < 0.06, medium 0.06–0.13, large ≥ 0.14) for the MANOVA and ANOVA analyses and as Cohen’s *d* (small < 0.50, medium < 0.80, large ≥ 0.80) for the pairwise comparisons [50].

#### Latent profile analysis (LPA)

Raw ATTEX-P scale scores were standardized according to the reference group to make the scales comparable to each other as well as to the level of typical development. Then, using Mplus 8.1 [51], models with different numbers of latent groups were fitted using the maximum likelihood method with robust standard errors as the estimation method. Only means were allowed to vary between groups. Different statistical criteria were considered when choosing the best-fitting model: Akaike’s information criteria (AIC) and Bayesian information criteria (BIC) are model evaluation criteria that take into account model fit and parsimony. The model with the lowest value is preferred. Vuong–Lo–Mendell–Rubin likelihood ratio (VLMR), Lo–Mendell–Rubin adjusted likelihood ratio (LMR), and bootstrap likelihood ratio test (BLRT) assess relative model fit by comparing a model with k groups to one with *k* − 1 groups, with a *p* value < 0.05 suggesting significant improvement in model fit. In addition, entropy, subgroup sample sizes, and overall model interpretability were evaluated when choosing the best model. Entropy is a standardized measure of the certainty of assigning participants into groups based on their model-derived posterior probabilities. The value of entropy ranges between 0 and 1, with higher values indicating clearer group delineation [52]. To ensure that the best log-likelihood value of each model did not reflect a local solution, 500 starting values were used, and the replication of the best log-likelihood was checked for each model. When interpreting the profiles, mean scores at or above the 90th percentile were considered to imply clinically significant impairment on a given scale, in accordance with the norms of ATTEX-P [46].

In the second phase, participants were assigned to groups based on their most likely profile membership. The relationship between group membership and background variables, including gender, age, and maternal education level, was examined via cross tabulation and $x^{2}$ tests. If the expected cell counts were less than 5 in 20% or more of the cells, exact tests were used. In addition, cross tabulation of the symptom groups with the person-oriented EF subgroups was performed to examine whether internalizing, externalizing, combined or mild symptoms were over- or underrepresented in the EF subgroups.

## Results

### Background characteristics of the symptom and reference groups

The means and standard deviations of the groups in demographic variables are displayed in Table 1. The groups differed significantly in gender ratio, *X*^*2*^(4) = 27.78, *p* < 0.001, with the COMB group including more boys than the reference group, *X*^*2*^(1) = 18.45, *p* < 0.001. The groups also differed in terms of age, *F*(4, 833) = 6.25, *p* < 0.001, $\eta_{\text{p}}^{2}$ = 0.03, with the COMB group including younger children than the reference group (*p* = 0.001). Although the groups also differed in maternal education level, *X*^*2*^(4) = 16.24, *p* = 0.003, significant differences in column proportions between specific symptom groups were not found after adjusting for 10 group comparisons.

### EF difficulties in the symptom and reference groups

Variables representing child’s age, gender, and maternal education level were included as covariates in all group comparisons. The groups differed from one another in the total EF score, *F*(4, 830) = 66.84, *p* < 0.001, $\eta_{\text{p}}^{2}$ = 0.24. Pairwise comparisons revealed that all symptom groups had a higher total score than the reference group, with all *p* values < 0.001 and *d* ranging between 0.91 and 1.66. No significant differences between the symptom groups in the total EF score were found; however, the effect size for the difference between the INT and the EXT groups was close to large (*d *= 0.75), indicating more EF problems overall in the EXT group (*M* = 46.19, SD = 12.86) than in the INT group (*M* = 29.38, SD = 20.92). The groups differed from one another also in the scale scores, Wilks’s lambda = 0.71, *F*(36, 3082) = 8.38, *p* < 0.001, $\eta_{\text{p}}^{2}$ = 0.08. ANCOVAs showed differences for each scale (Table 2). Pairwise comparisons revealed that all of the symptom groups had higher scores than the reference group on eight of the nine scales (*p* values ranging between < 0.001 and 0.039, *d* ranging between 0.66 and 1.68). On the motor hyperactivity scale, the difference between the REF and INT groups was not significant (*p* = 0.066, *d *= 0.56). The symptom groups differed from one another on impulsivity, with the INT group having a lower score than the EXT (*p* = 0.009, *d *= 1.00), COMB (*p* = 0.035, *d *= 0.70), and MILD (*p* = 0.030, *d *= 0.71) groups. On motor hyperactivity, the INT group had a lower score than the EXT (*p* = 0.003, *d *= 1.09) and COMB (*p* = 0.036, *d *= 0.70) groups. Of the insignificant differences between the INT and the EXT groups, the effect sizes for distractibility (*d *= 0.79), execution of action (*d *= 0.75), and sustaining attention (*d *= 0.69) were substantial, indicating more EF problems in the EXT than in the INT group in these domains. On the remaining scales (directing attention, shifting attention, initiation, and planning), the effect sizes for the insignificant differences between the INT and the EXT groups were small, with *d* ranging between 0.07 and 0.26.

**Table 2:** Means (standard deviations), estimated marginal means (standard errors) and ANCOVA results for the symptom groups and the reference group on the ATTEX-P scales

EF scale	INT (*n* = 24)		EXT (*n* = 21)		COMB (*n* = 6)		MILD (*n* = 66)		REF (*n* = 667)		*F*(4, 830)	*p*^a^	$\eta_{\text{p}}^{2}$
	*M* (SD)	EMM (SE)	*M* (SD)	EMM (SE)	*M* (SD)	EMM (SE)	*M* (SD)	EMM (SE)	*M* (SD)	EMM (SE)			
Distractibility	4.32 (2.87)	4.30 (0.51)	6.86 (2.13)	6.28 (0.55)	6.03 (2.51)	5.46 (0.33)	5.49 (3.18)	5.19 (0.31)	1.92 (2.60)	2.02 (0.10)	56.00	< 0.001	0.21
Impulsivity	6.53 (6.14)	6.54 (0.98)	12.24 (4.31)	11.33 (1.05)	10.80 (6.04)	9.93 (0.63)	10.40 (6.73)	9.94 (0.59)	3.17 (4.63)	3.32 (0.19)	58.44	< 0.001	0.22
Motor hyperactivity	2.67 (2.87)	2.66 (0.49)	5.71 (2.33)	5.27 (0.52)	4.78 (3.21)	4.35 (0.31)	4.30 (3.62)	4.07 (0.30)	1.23 (2.23)	1.31 (0.09)	47.67	< 0.001	0.19
Directing attention	3.42 (2.70)	3.42 (0.46)	4.10 (2.49)	3.71 (0.50)	3.85 (2.54)	3.46 (0.30)	3.98 (2.83)	3.76 (0.28)	1.56 (2.24)	1.63 (0.09)	24.43	< 0.001	0.10
Sustaining attention	2.54 (2.25)	2.56 (0.40)	4.10 (2.49)	3.90 (0.42)	4.08 (2.44)	3.71 (0.25)	3.65 (2.76)	3.45 (0.24)	1.04 (1.88)	1.10 (0.08)	49.43	< 0.001	0.19
Shifting attention	2.86 (2.40)	2.85 (0.41)	3.71 (2.12)	3.38 (0.44)	4.07 (2.46)	3.73 (0.26)	3.76 (2.71)	3.57 (0.25)	1.22 (1.93)	1.28 (0.08)	40.14	< 0.001	0.16
Initiative	2.13 (1.90)	2.10 (0.32)	2.19 (1.57)	1.99 (0.34)	2.91 (1.99)	2.69 (0.20)	2.47 (2.08)	2.34 (0.19)	0.95 (1.47)	0.99 (0.06)	27.05	< 0.001	0.12
Planning	1.88 (1.80)	1.90 (0.28)	2.29 (1.85)	2.02 (0.30)	2.65 (1.96)	2.40 (0.18)	2.54 (1.83)	2.39 (0.17)	0.69 (1.30)	0.74 (0.05)	40.43	< 0.001	0.16
Execution of action	3.04 (2.54)	3.03 (0.39)	4.81 (1.66)	4.45 (0.42)	4.30 (2.59)	3.94 (0.25)	4.09 (2.72)	3.89 (0.24)	1.20 (1.80)	1.26 (0.07)	60.58	< 0.001	0.23
Total score	29.38 (20.92)	29.35 (3.51)	46.19 (12.86)	42.33 (3.76)	43.47 (20.05)	39.68 (2.25)	40.69 (23.90)	38.59 (2.12)	12.98 (17.18)	13.65 (0.67)	66.84	< 0.001	0.24

Child’s age, gender and maternal education level were included as covariates in all analyses

*INT* children with internalizing symptoms, *EXT* children with externalizing symptoms, *COMB* children with combined symptoms, *MILD* children with mild symptoms, *REF* reference children

Higher scores on the scales indicate more problems

^a^Significant results of the pairwise comparisons for total score, distractibility, impulsivity, directing attention, sustaining attention, shifting attention, initiative, planning and execution of action: REF < EXT, COMB, MILD, INT; for motor hyperactivity: REF < EXT, COMB, MILD. The symptom groups differed from one another on impulsivity (INT < EXT, COMB, MILD) and on motor hyperactivity (INT < EXT, COMB)

### EF difficulties in the person-oriented subgroups

A summary of LPA model fit indices is presented in Table S1 in Supplementary Materials. The BIC reached its lowest value in a five-group model, indicating an optimal solution. The AIC suggested six or more groups to be the preferred solution. Out of the comparative model fit indices, the BLRT suggested each consecutive model above a one-group model to provide a significant improvement in fit. The LMR and the VLMR indicated that four would be the maximum number of groups to consider. Thus, the statistical criteria gave support for models with four, five, and six groups. We rejected the six-group solution, because the sample size was too small in one subgroup (*n* = 9) and due to problems with interpretation. Both the four- and five-group solutions had adequate sample sizes in each subgroup as well as high entropy (0.93 and 0.92, respectively). We decided to prioritize the BIC, because it has been shown to perform better in the case of a small overall sample size and continuous indicator variables [53]. In addition, further analyses relating psychiatric symptoms to group membership provided support for the external validity of both the groups that were merged into one in the four-group model. Therefore, we chose the five-group model.

**Fig. 1:**
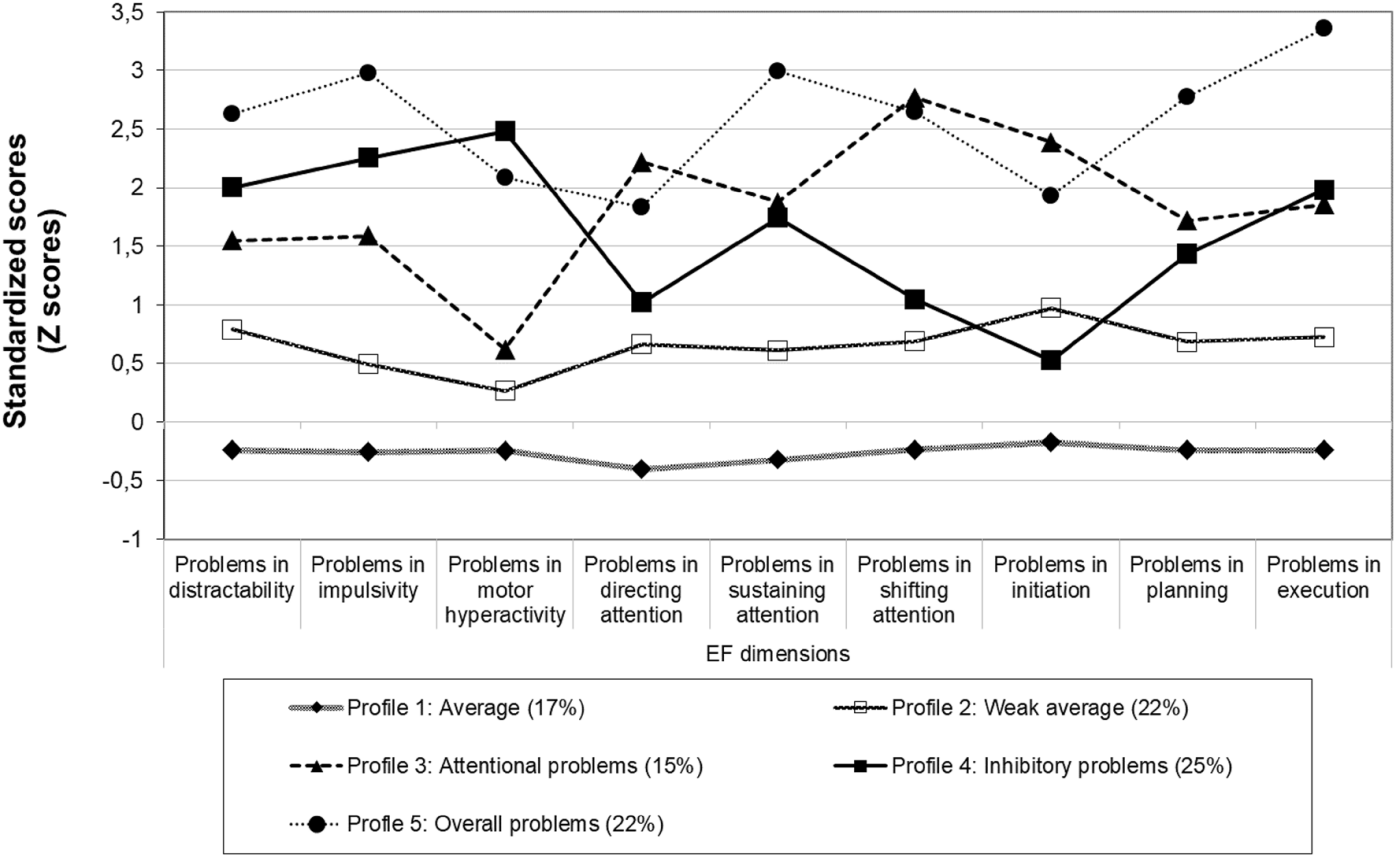
EF profiles of the five subgroups identified via latent profile analysis. Higher scores on the scales indicate more problems

EF profiles of the five obtained subgroups groups are shown in Fig. 1. The first group (*n* = 29, 17%), named average, had average EF abilities across all domains. On no indicator did this group perform worse than the reference group, and in directing attention, their performance was nearly half a standard deviation below the reference group (i.e., their performance was better). The second group (*n* = 37, 22%) had slightly below average abilities on all EF indicators and was named weak average. Despite a mild elevation on the Initiation scale (0.97 standard deviations above the typical level), they did not have clinically relevant impairment in any EF domain. The third group (*n* = 25, 15%) had clinically relevant deficits in all EF domains except in motor hyperactivity. Due to not having high motor hyperactivity but showing severe deficits in attention-related domains, especially in shifting attention, this group was named attentional problems. The fourth group (*n* = 42, 25%) exhibited a profile that was somewhat of a mirror image to the third group. These children had particularly high motor hyperactivity, but showed no clinically significant deficits in directing or shifting attention nor in initiating behavior and was named inhibitory problems. The fifth group (*n* = 38, 22%) had clinically relevant and severe deficits across all EF domains and was thus named overall problems.

Significant mean differences between the groups on the EF scale scores were found, Wilks’s lambda = 0.03, *F*(36, 593) = 27.48, *p* = < 0.001, $\eta_{\text{p}}^{2}$ = 0.60. As presented in Table S2 in the Supplementary Materials, the groups differed significantly from one another on all scales.

### Background characteristics and psychiatric symptoms in the person-oriented subgroups

The results of the cross tabulation of the EF subgroups and background characteristics are presented in Table 3. The EF subgroups did not differ in terms of age, *F*(4, 166) = 0.60, *p* = .595, $\eta_{\text{p}}^{2}$ = 0.02. A significant association between group membership and gender was found, *X*^*2*^(4) = 31.14, *p* < .001. The adjusted residuals suggested that there were more boys than expected in the inhibitory problem group (adj. res. = 3.2), and more girls than expected in the average (adj. res. = 4.4) and weak average (adj. res. = 2.2) groups. The groups also differed in terms of maternal education level, *X*^2^(4) = 15.63, *p* = .004. Low maternal education was overrepresented in the inhibitory problem group (adj. res. = 2.6) as compared to the other groups, and high maternal education was overrepresented in the average and weak average groups (adj. res. = 2.3 and 2.2, respectively).

**Table 3:** Distribution of child and contextual characteristics in the person-oriented EF subgroups

	Average	Weak average	Attentional problems	Inhibitory problems	Overall problems
*N*	29	37	25	42	38
Age in years^a^, *M* (SD)	5.7 (0.7)	5.7 (0.7)	5.9 (0.6)	5.7 (0.7)	5.7 (0.8)
Gender					
Boys (%)	11 (9.0; 37.9)	21 (17.2; 56.8)	21 (17.2; 84,0)	38 (31.1; 90.5)	31 (25.4; 81.6)
Adj. res.	**− 4.4**	**− 2.2**	1.5	**3.2**	1.6
Girls (%)	16 (36.7; 62.1)	16 (32.7; 43.2)	4 (8.2; 16.0)	4 (8.2; 9.5)	7 (14.3; 18.4)
Adj. res.	**4.4**	**2.2**	− 1.5	**− 3.2**	− 1.6
Maternal education					
Low (%)	10 (10.9; 34.5)	14 (15.2; 37.8)	13 (14.1; 52.0)	30 (32.6; 71.4)	25 (27.2; 65.8)
Adj. res.	**− 2.3**	**− 2.2**	− 0.2	**2.6**	1.7
High (%)	19 (24.1; 65.5)	23 (29.1; 62.2)	12 (15.2; 48.0)	12 (15.2; 28.6)	13 (16.5; 34.2)
Adj. res.	**2.3**	**2.2**	0.2	**− 2.6**	− 1.7
Symptom group					
INT (%)	6 (25.0; 20.7)	10 (41.7; 27.0)	3 (12.5; 12.0)	2 (8.3; 4.8)	3 (12.5; 7.9)
Adj. res.	1.1	**2.6**	− 0.3	**− 2.0**	− 1.2
EXT (%)	0 (0.0; 0.0)	4 (19.0; 10.8)	1 (4.8; 4.0)	12 (57.1; 28.6)	4 (19.0; 10.5)
Adj. res.	**− 2.2**	− 0.3	− 1.4	**3.7**	− 0.4
COMB (%)	7 (11.7; 24.1)	13 (21.7; 35.1)	11 (18.3; 44.0)	15 (25.0; 35.7)	14 (23.3; 36.8)
Adj. res.	− 1.4	0.0	1.0	0.1	0.3
MILD (%)	16 (24.2; 55.2)	10 (15.2; 27.0)	10 (15.2; 40.0)	13 (19.7; 31.0)	17 (25.8; 44.7)
Adj. res.	**2.0**	− 1.6	0.2	− 1.2	0.9

Adjusted residuals (adj. res.) that have an absolute value over 1.96 (bolded in the table) are considered significant

Percentages are expressed as within row; within column

*INT* children with internalizing symptoms, *EXT* children with externalizing symptoms, *COMB* children with combined symptoms, *MILD* children with mild symptoms, *REF* reference children

^a^Group differences in mean age were non-significant

Cross tabulation of the EF and symptom groups was conducted to examine whether clinically significant levels of internalizing, externalizing, combined, or mild symptoms would be over- or underrepresented in certain EF subgroups (Table 3). Differences between the EF subgroups in symptom type were found (*p* = 0.005, Fisher’s exact test). Externalizing symptoms were overrepresented in the inhibitory problem group (adj. res. = 3.7), and internalizing symptoms were overrepresented in the weak average group (adj. res. = 2.6), and mild symptoms were overrepresented in the average group (adj. res. = 2.0).

## Discussion

The purpose of this study was to investigate EFs among preschoolers with psychiatric symptoms. First, we followed a traditional approach of comparing children classified into groups based on their level of internalizing and externalizing symptoms. Groups of children with internalizing, externalizing, combined, or mild symptoms were compared to a reference group and to one another on the ATTEX-P total and scale scores. Second, we further examined the heterogeneity of EFs within the clinical sample using a person-oriented approach of empirically identifying subgroups of children showing distinct EF profiles. Associations between the subgroups and different indicators, including gender, age, maternal education, and psychiatric symptoms, were then examined to understand differences between the subgroups also beyond EFs.

When controlling for gender, age, and maternal education, all of the symptom groups differed from the reference group in nearly all EF domains, suggesting that, overall, young psychiatric outpatients tend to demonstrate poorer EF abilities than their typically developing peers regardless of their type of emotional and behavioral symptoms. The broad EF problems of the preschoolers with mainly externalizing and both externalizing and internalizing symptoms were in accordance with our hypotheses and similar to previous studies using EF rating scales [30, 32, 33, 36]. In addition, in accordance with our hypotheses, the children with internalizing symptoms had more problems in shifting attention than those in the reference group. However, they had more problems than the reference group in nearly all other EF domains as well, indicating that their EF difficulties in the day care environment were widespread. In motor hyperactivity, the difference was not significant, yet the moderate effect size suggests that children with internalizing symptoms may have more problems with hyperactivity than children in general do. The findings concerning children with internalizing symptoms resemble those of Skogan et al. [36], who discovered that anxious preschoolers scored higher than reference children on all scales of the BRIEF-P. In addition, in accordance with our findings, Cataldo et al. [54] found increased levels of behavioral impulsivity in a clinical sample of school-aged depressed children. In contrast to our findings, Eisenberg et al. [32] found, in a normative sample, that children with internalizing symptoms were rated as less impulsive than controls were and concluded that these children seem to exhibit an “overcontrolled” style of regulation. The fact that the children with internalizing symptoms in our clinical sample were rated as more impulsive than the reference children, albeit to a lesser degree than the children with externalizing, combined, or even mild symptoms, could reflect differences in samples (clinical vs. normative). Somewhat different patterns of everyday EFs may be expected for children with internalizing symptoms within clinical and non-clinical settings, particularly in terms of impulsivity, highlighting the need to study the relationship between EFs and internalizing symptoms at different levels of symptom severity and comorbidity.

Differences between the symptom groups emerged in impulsivity and motor hyperactivity. The children with internalizing symptoms showed less problems in these aspects of EFs than other children with psychiatric symptoms, and as expected, the difference was most substantial between the internalizing and externalizing groups. The substantial effect sizes for differences between the internalizing and externalizing groups in distractibility and execution of action may indicate that differences in these EF domains exist as well, with the children high in externalizing symptoms having more problems. In accordance with the findings of Eisenberg et al. [32], the children with both internalizing and externalizing symptoms had similar EF difficulties as the children with mainly externalizing symptoms. Thus, high levels of combined symptoms do not seem to make children more or less prone to EF difficulties than having high externalizing symptoms only.

Apart from the differences in impulsivity and motor hyperactivity, the symptom groups had similar difficulties in most EF domains, suggesting that clinically referred children have more similarities than differences in terms of EF behaviors. However, it could also indicate that the classification of children based on their symptoms did not ideally capture the full heterogeneity of EFs present in the sample. By further investigating the latter option using LPA, five profiles were discerned, with one group of children showing average EF behaviors, one group showing weak average EF behaviors, and three groups showing major EF difficulties with either attentional problems, inhibitory problems, or problems in all aspects of the EFs evident (Fig. 1). The identification of qualitatively different subgroups implies that, in addition to the high overall rates of EF impairment present among young child psychiatric outpatients [9], considerable heterogeneity also exists. Importantly, examining individual-level differences in EFs seemed to provide more fine-grained information than did the comparisons of internalizing/externalizing groups. The person-oriented approach seemed to better display inter child differences in multiple different domains of EFs, such as in attentional functions, initiating action, and planning.

The finding that the subgroups differed not only in the severity, but also in the pattern of difficulties is in contrast with the findings of Dajani et al. [43], who identified only severity differences in the EF profiles of children with neurodevelopmental disorders. Importantly, a portion of the children—those belonging to the average and weak average groups—did not demonstrate clinically relevant impairment in any EF domain. Likewise, Kavanaugh et al. [42] reported that 68% of their sample of child psychiatric inpatients displayed neurocognitive impairment. They concluded that neurocognitive weaknesses are not present in all children with severe psychiatric disorders. Similarly, among preschool-aged psychiatric outpatients, a notable subgroup does not seem to display clinically significant EF impairment in the day care context.

In addition to not showing clinically significant EF impairment, the children with an average EF profile were characterized by mild psychiatric symptoms (below clinical levels of both internalizing and externalizing symptoms). The reason for the psychiatric referral of these children could be primarily related to other problems than the child’s behavior, e.g., crisis in the family or parenting issues. A weak average profile was associated with clinically significant levels of internalizing symptoms (with problems in initiation slightly standing out). A profile marked by inhibitory problems, evident as high levels of impulsivity, distractibility, and hyperactivity, was associated with clinically significant levels of externalizing symptoms, which is in accordance with the previous literature suggesting that children with externalizing symptoms have particular problems with respect to inhibition [31, 33, 36].

Neither high nor low internalizing and/or externalizing symptoms were related to the profiles marked by attentional or overall problems. The children showing these profiles may have psychiatric symptoms that are not well captured by the internalizing/externalizing domains of the CBCL, e.g., attentional symptoms related to the inattentive subtype (ADHD-I) and/or social and communicative symptoms characteristic of autism spectrum problems. Among school-aged children, inattention and autism-related problems have been considered as separate domains of psychopathology alongside with internalizing, externalizing, and non-specific domains [55]. Previous literature suggests that children with ADHD-I tend to have difficulties in many aspects of EFs, but less in response inhibition [56, 57], similar to the pattern of EFs shown by the attentional problem group. This group was also the most impaired in shifting attention, and deficits in set shifting or cognitive flexibility have been associated with autism spectrum problems [58, 59]. In addition, the profile marked by severe overall EF problems may be more related to the severity and chronicity of psychiatric symptoms than to any specific symptom type per se [34]. Similar EF problems have previously been indicated in children with both inattentiveness and hyperactivity [57] as well as autism spectrum problems [60, 61], and in comorbid groups [43].

In addition to psychiatric symptom type, EF subgroup membership was significantly associated with gender and maternal education level. The group with inhibitory problems was characterized by low maternal education and a high prevalence of boys. The groups with average and weak average EF profiles were characterized by a high prevalence of girls and high maternal education. This is in accordance with the previous findings suggesting that, in the preschool period, boys tend to display more EF difficulties than girls do [13, 14]. In addition, the previous studies have linked higher parental education to better EF abilities in children [14, 15]. Our findings indicate that low maternal education is particularly pronounced in a subgroup of children showing inhibitory problems and not necessarily in the subgroup showing the highest overall levels of EF impairment. Externalizing symptoms were also pronounced in the inhibitory problem group, thus underlining the existence of a subgroup of preschoolers among whom cognitive, socioemotional, and environmental risk factors tend to accumulate.

The relationship between psychopathology and EF difficulties has been studied in methodologically diverse ways, which may explain some of the variability in results and make comparisons between studies difficult. In terms of the present study, it should be kept in mind that rating scales generally show only low-to-moderate correlations with performance-based measures, highlighting the fact that they tap somewhat different underlying constructs [62]. Ideally, future studies should utilize both performance-based measures and rating scales to validate the present findings. In addition, children’s emotional and behavioral problems were evaluated by parents and EF behaviors by day care teachers. It is widely known that raters across different situations generally have low agreement [63], as different environments (day care, home) have different expectations and bring out different aspects of the child’s behavior. Parents may be at an advantage to evaluate their children’s internalizing problems, because these problems may not come out so easily in the day care or school environment [64]. In addition, teacher ratings of children’s EFs might tap an aspect of the EF construct that has particular bearings on important school-related outcomes [65]. Overall, the utilization of different informants to report on different aspects of children’s behavior can be seen as a strength of the present study, as it eliminates the possibility that the results would be due to same rater bias.

Some other limitations should also be noted. First, some overlap between the rating scale items measuring externalizing symptoms and EFs—mainly impulsivity, motor hyperactivity, and sustaining attention—exist and can artificially magnify the relationship between externalizing symptoms and the mentioned EF behaviors. Although the overlap is small, as the externalizing problems scale of the CBCL is mostly comprised of items assessing aggressive behavior, it should be taken into account when interpreting the results. Second, small symptom group sizes reduced the power to find significant effects, and therefore, effect sizes were examined in addition to *p* values for all pairwise comparisons. The clinical sample as a whole was also somewhat small for LPA. Replications of the group solution with larger clinical samples are needed to justify the existence of the subgroups identified here. Finally, the cross-sectional nature of the present study does not allow any conclusions to be drawn about the direction of relationships. It remains to be investigated whether primary EF problems can place a child at risk for the development of psychiatric problems or the other way around, or whether the two kinds of problems reflect a common underlying vulnerability and thus often coexist.

A strength of the present study was its utilization of two complementary methodological approaches. Despite providing useful information on a group level, a drawback of the variable-oriented approach is its assumption of uniformity of the groups. For instance, the externalizing group may include children with very different kinds of symptoms, as some may have problems related to aggressive behavior and others mainly to hyperactivity. Thus, the EF profiles of these children may markedly differ from one another. In this study, the person-oriented approach was useful in revealing such heterogeneity within the internalizing/externalizing groups. For instance, although the internalizing group showed more EF problems overall than the reference group, the majority of the children with internalizing symptoms had average or close to average EF behaviors in all domains. However, approximately one-third had severe problems, and in psychiatric care, the identification of these children via screening is important. In addition, a benefit of the person-oriented approach is its ability to find underlying EF subgroups that may not correspond to any known diagnostic or symptom groups. If future studies validate these subgroups, EF interventions specifically targeted at children with matching EF profiles could be designed.

The present findings suggest that clinically referred preschool children, regardless of the type of psychiatric symptoms they have, tend to display more everyday EF problems than typically developing children do. Children with internalizing symptoms tend to have less difficulties in inhibiting undesirable behaviors than other children with psychiatric symptoms do, but beyond that, the diagnostic groups show little difference. Heterogeneity in other EF behaviors, including attention-related functions, planning and acting on one’s own initiative becomes apparent when EF profiles are identified based on individual variation in EFs. Clinically, the present findings imply that the screening of EF difficulties is important regardless of a child’s psychiatric symptoms. In case signs of EF difficulties arise, a comprehensive evaluation of the child’s EF profile is important, so that the EF strengths and weaknesses may be identified and considered when planning for intervention.

## Electronic supplementary material

Below is the link to the electronic supplementary material.

Supplementary material 1 (DOCX 18 kb)
