# Supplementary material for: Heterogeneity of executive functions among preschool children with psychiatric symptoms
Source: Eur Child Adolesc Psychiatry. 2019 Nov 11;29(9):1237–49. doi: 10.1007/s00787-019-01437-y (PMC7497399; doi:10.1007/s00787-019-01437-y)
Supplement: Supplementary file 1 — Supplementary material 1 (DOCX 18 kb) [file 787_2019_1437_MOESM1_ESM.docx]

**Heterogeneity of executive functions among preschool children with psychiatric symptoms**

Sini Teivaanmäki, Hanna Huhdanpää, Noona Kiuru, Eeva T. Aronen, Vesa Närhi, & Liisa Klenberg

Submitted to: European Child & Adolescent Psychiatry

Corresponding author: Sini Teivaanmäki, Department of Psychology, University of Jyväskylä, Jyväskylä, Finland.

Email:sini.teivaanmaki@nmi.fi

SUPPLEMENTARY TABLES

| **Table S1.** Fit indices and class frequencies for latent profile analyses with different numbers of latent profiles for EF domains. | | | | | | | | |
| --- | --- | --- | --- | --- | --- | --- | --- | --- |
| No. of groups | BIC | aBIC | AIC | Entropy | *p* value of LMR | *p* value of VLMR | *p* value of BLRT |  |
| 1 (N=171) | 5325.05 | 5268.05 | 5268.50 |  |  |  |  |  |
| 2 (n1 = 66, n2 = 105) | 4609.47 | 4520.81 | 4521.50 | .96 | < .001 | < .001 | < .001 |  |
| 3 (n1= 59, n2 = 44, n3 = 68) | 4441.28 | 4320.95 | 4321.89 | .93 | .009 | .001 | < .001 |  |
| 4 (n1 = 45, n2 = 54, n3 = 32, n4 = 40) | 4363.74 | 4211.75 | 4212.94 | .93 | .0560 | .0530 | < .001 |  |
| **5 (n1 = 29, n2 = 37, n3 = 25, n4 = 42, n5 = 38)** | **4319.19** | **4135.53** | **4136.97** | **.92** | **.4003** | **.3942** | **< .001** |  |
| 6 (n1 = 35, n2 = 28, n3 = 16, n4 = 9, n5 = 44, n6 = 39) | 4332.74 | 4117.42 | 4119.10 | .93 | .4327 | .4298 | < .001 |  |
| *Note.* BIC = Bayesian Information Criterion; aBIC = Adjusted Bayesian Information Criterion; AIC = Akaike’s Information Criterion; LMR = Lo-Mendell-Rubin Adjusted Likelihood Ratio Test; VLMR = Vuong-Lo-Mendell-Rubin Likelihood Ratio Test; BLRT = Bootstrapped Likelihood Ratio Test. | | | | | | | | |

| **Table S2**. Standardized^a^ EF scale score means, standard deviations and ANOVA results for the person-oriented EF subgroups. | | | | | | | | |
| --- | --- | --- | --- | --- | --- | --- | --- | --- |
| EF scale | Average | Weak  average | Attentional  problems | Inhibitory  problems | Overall  problems | *F*(4,166) | *p* | ${}_{p}^{2}$ |
| Distractibility | -.24 (.48) | .76 (.49) | 1.55 (.46) | 2.02 (.53) | 2.62 (.42) | 180.08 | < .001 | .81 |
| Impulsivity | -.26 (.41) | .48 (.65) | 1.60 (1.01) | 2.24 (.66) | 2.98 (.63) | 127.04 | < .001 | .75 |
| Motor hyperactivity | -.24 (.35) | .28 (.85) | .62 (.69) | 2.50 (.70) | 3.08 (.64) | 160.96 | < .001 | .80 |
| Directing attention | -.40 (.40) | .66 (.85) | 2.14 (1.19) | 1.00 (.93) | 1.82 (.83) | 39.50 | < .001 | .49 |
| Sustaining attention | -.34 (.39) | .62 (.97) | 1.84 (.99) | 1.72 (.72) | 2.99 (.66) | 89.70 | < .001 | .68 |
| Shifting attention | -.24 (.48) | .65 (.80) | 2.73 (.75) | .99 (.87) | 2.64 (.72) | 92.31 | < .001 | .69 |
| Initiation | -.17 (.70) | .92 (1.20) | 2.37 (.96) | .49 (1.02) | 1.91 (1.09) | 30.65 | < .001 | .43 |
| Planning | -.25 (.55) | .69 (1.09) | 1.73 (1.15) | 1.40 (1.12) | 2.75 (1.00) | 40.89 | < .001 | .50 |
| Execution | -.26 (.51) | .74 (.72) | 1.82 (.85) | 1.96 (.83) | 3.35 (.81) | 107.92 | < .001 | .72 |
| Total score | -.31 (.28) | .73 (.31) | 2.07 (.48) | 2.04 (.37) | 3.21 (.40) | 451.76 | < .001 | .92 |
| *Note.* Higher scores on the scales indicate more problems.  ^a^Z-scores are in reference to the normative group. | | | | | | | | |
